# Lingering challenges in everyday life for adults under age 60 with hip fractures – a qualitative study of the lived experience during the first three years

**DOI:** 10.1080/17482631.2023.2191426

**Published:** 2023-03-17

**Authors:** Sebastian Strøm Rönnquist, Hilda K Svensson, Charlotte Myhre Jensen, Søren Overgaard, Cecilia Rogmark

**Affiliations:** a Department of Orthopaedics, Lund University, Skåne University Hospital, Malmö, Sweden; b Department of Orthopaedic Surgery and Traumatology, Odense University Hospital, Odense, Denmark; c Academy of Health and Welfare and Centre of research on Welfare, Health and Sport (CVHI), Halmstad University, Halmstad, Sweden; d Department of Clinical Research, University of Southern Denmark, Odense, Denmark; e Department of Orthopaedic Surgery and Traumatology, Copenhagen University Hospital – Bispebjerg and Frederiksberg, Copenhagen, Denmark; f Department of Clinical Medicine, Faculty of Health and Medical Sciences, University of Copenhagen, Copenhagen, Denmark

**Keywords:** Hip fracture, young and middle-aged adults, recovery, experience, everyday life

## Abstract

**Purpose:**

We aimed to illuminate the lived experiences and the path of recovery for adults sustaining a hip fracture before the age of 60.

**Methods:**

Participants were purposively sampled from a prospective multicenter cohort study in Sweden and Denmark, and narrative interviews were conducted with 19 individuals 0.7–3.5 years after the fracture. We used a phenomenological hermeneutic method to describe the participants’ expressed essential meaning.

**Results:**

The experience of sustaining a hip fracture was expressed as a painful and protracted process of regaining self-confidence, function, and independence. It also implied a sense of growing old from one day to the next. Participants were afraid of new falls and fractures, resulting in an increased wariness. When expressing fears and persisting symptoms, participants described being neglected and marginalized by the healthcare system, which was perceived as non-receptive and routinely driven by a notion that hip fractures affect only the elderly. Rehabilitation targeted towards needs different from those of elderly individuals was requested.

**Conclusion:**

The lived experience of sustaining a hip fracture in individuals under 60 includes substantial challenges in everyday life, even up to 3.5 years after the injury. Rehabilitation pathways tailored to the needs of younger patients are requested.

## Background

An individual with a hip fracture is typically seen as old and frail, and is assumed to have a fracture caused by low-energy trauma (Bertram et al., 2011). Incurring a hip fracture at an older age is associated with an increased risk of functional deficit, persisting pain, increased fear of falling, decreased health-related quality of life and death (Bertram et al., 2011; Jellesmark et al., 2012; Sale et al., 2017). Strategies such as remaining active, managing expectations and maintaining participation in activities have been described as essential to maintain function and quality-of-life (Sims-Gould et al., 2017). Is this also the case for young and middle-aged adults sustaining hip fractures? This more heterogeneous group constitutes less than one tenth of all hip fractures (Farooq et al., 2005; Karantana et al., 2011; Omari et al., 2019; Robinson et al., 1995; Strøm Rönnquist et al., 2022; Wang et al., 2017). While some are healthy, others are predisposed to fractures due to lifestyle factors, functional limitations, hormonal deficiency or diseases (Karantana et al., 2011; Rogmark et al., 2018; Strøm Rönnquist et al., 2022). Among older adults, we know that empowerment of patients was not adequately achieved in the hip fracture pathways (Jensen et al., 2017). However, there is a lack of knowledge regarding younger patients’ perspectives. The need for increased awareness of their experiences of sustaining a hip fracture and the subsequent recovery led us to conduct this study, to be able to design better future fracture management and rehabilitation. Our aim was to illuminate the lived experiences and the path of recovery for adults sustaining a hip fracture before the age of 60. A clinical perspective was to involve patients’ experiences in future healthcare management to ensure that their needs are effectively addressed.

## Methods

### Study design

The present work is a qualitative study using a phenomenological hermeneutic method described by Lindseth and Norberg (Lindseth & Norberg, 2004). This qualitative method was chosen to build a deeper understanding of the expressed lived experiences of individuals sustaining hip fractures before the age of 60 based on their narratives. In the reporting of this study, the Standards for Reporting Qualitative Research (O’brien et al., 2014) were considered.

### Setting and sampling

Participants were purposively sampled from the prospective, multicenter cohort study, *Hip fractures in adults under the age of 60 years (HFU-60)*, which analyzes the epidemiology, treatment and outcome of hip fractures (Strøm Rönnquist et al., 2022). Individuals aged 18–59 years, who sustained a hip fracture and were treated at any of the four participating orthopaedic departments in public hospitals in Southern Scandinavia, were eligible for inclusion in the cohort study regardless of medical, cognitive, and functional status prior to the fracture. Patients with pathological fractures, non-acute fractures, or not residing in the catchment areas were excluded from study participation. Fracture treatment and support of recovery were essentially similar and provided as per the standard regimens of the respective departments, no other treatment was provided within the frames of the study. From the total cohort of 218 participants in the HFU-60 study, 30 participants from Malmö and Odense, representing a variety of characteristics, were invited in early 2019 by mail and phone by the two authors performing the interviews. The inclusion criteria used for the purposive sampling were as follows: speaking Swedish or Danish, being able to individually partake in the interview, and minimum 6 months’ time since the hip fracture. At the time of inclusion, the participants were provided with written and oral information on the study; regarding the purpose, the form of the interview, and written consent was collected.

### Ethical considerations

The study was conducted in accordance with the Helsinki Declaration, and all participants provided informed written consent. Approval was obtained from ethical review boards in Sweden (Regionala etikprövningsnämnden Lund (dnr: 2015/28)) and Denmark (the Regional Health Service and University Research Ethics Committee and the Danish Data Agency (S-20150137) (case approval no 15/51398)). Data was pseudonymized and stored in a database, only accessible by the authors. All quotations from participants were included with permission and are coded according to country and participant number, e.g., [D2]. Data may be made available upon reasonable request to the corresponding author.

### Data collection

Participants were interviewed in either their homes (*N* = 16) or a hospital outpatient setting (*N* = 2), based on preference. One participant preferred to be interviewed by telephone, all other interviews were one-to-one physical interviews. The interviews were conducted from April to August 2019, the median time from the hip fracture to the interview was 1.5 years (interquartile range (IQR) 1.3–3). The interviews lasted between 35 and 71 minutes;, data collection in the individual interviews continued until data saturation was obtained; i.e., no new aspects or experiences presented themselves in the interviews. Basic demographic data (age, marital status, occupation, comorbidity, previous fractures) and history of the present hip fracture incident were collected before the interviews. The interviews were initiated with an open-ended question: “Could you tell me about when you sustained your hip fracture and how you have experienced the time after as well as your recovery?”. A complementary interview guide was used by the interviewer, with follow-up questions such as, “How was the first time-period when you came home from the hospital?”, “Do you have any symptoms from your hip today?”, “Can you describe your feelings towards your fracture?”, and “What is your opinion of the care that you received both at the hospital but also once you were discharged?”. The follow-up questions were intended to keep the interviewee narrate within the aim of the study.

### Researcher characteristics and reflexivity

The research team represented different fields within both qualitative and quantitative areas of research. The authors have previous experience in fracture research, with specific expertise in hip fracture research, predominantly represented by the last author. The interviews were conducted by two experienced qualitative researchers, HKS and CMJ, in the interviewers’ and the respondents’ native language (Swedish and Danish, respectively). The interviewers were not involved in the fracture treatment, hospital care or rehabilitation. The recorded data material was transcribed by the interviewer in the language in which the interview was conducted. For a joint analysis on both datasets, we performed triangulation during data collection through comparisons by the bilingual author SSR, who evaluated whether the two national data collections were conducted in a comparable way, to ensure that the interviews were performed similarly, without systematic differences. Trustworthiness was established by demonstrating reflexivity, credibility, transferability, and dependability according to Koch’s (Koch, 2006) criteria (Table I).

**Table I.** Demonstrating trustworthiness in the qualitative data collection and analysis (Ellingsen et al., 2015; Koch, 2006; Lincoln & Guba, 1985; Miles & Huberman, 1994; Polit & Hungler, 1999; Sandelowski, 1986).

Trustworthiness criteria	Fulfilment of criteria
Reflexivity	Data, themes, and saturation of findings were continuously discussed amongst the analyzing authors. The analyzing authors were also responsible for the interviews and collection of data, adding tacit knowledge and a more profound understanding. To understand the impact of and on our pre-understanding of the narratives, as well as to grasp potential decisive parts of the narration, participants were asked elaborating questions. Field notes regarding context, thoughts, and description of the location were collected to give the narration a contextualized frame.
Credibility	Findings were based on participants’ narratives. Both interviewers and a bilingual author were involved in the process of analysis to establish consistency and researcher triangulation in the interpretation of the data.
Transferability	By using a purposive sampling frame and recruiting participants representing different demographic characteristics in form of marital status, level of education, employment, comorbidity and cause of hip fracture, the experiences from a broad spectrum of patients were enlightened. Participants in the current study were sampled from a larger prospective mixed general population cohort of adults under the age of 60 with hip fractures from two Scandinavian countries with public healthcare (Strøm Rönnquist et al., 2022). The participating departments are the only emergency hospitals in their respective catchment area and treat all individuals presenting with hip fractures. The cohort comprised three quarters of all patients under the age of 60 treated for a hip fracture during the study inclusion time, reflecting the heterogeneity in this patient group.
Dependability	Findings were continuously evaluated and challenged in iterative processes, by holding regular team meetings throughout the data collection and analysis periods.

### Data analysis

The interpretation using the method of phenomenological hermeneutics was conducted on 3 levels (Lindseth & Norberg, 2004; Strandberg et al., 2001).

1. Naïve reading involved reading the text several times as openly as possible to obtain a general understanding of the meaning behind the words, rather than what the participants said. Each interviewer constructed a naïve understanding of their interview data, which were translated into English early on. In the comparison of naïve readings, we found that findings in the Swedish and Danish interviews were echoed in one another, enabling a joint analysis of data. This superficial deduction provided direction for the next level of interpretation.
2. In the structural analysis, themes were developed through interpretation of the interviews via three levels of understanding (Supplement Table S1). Data was sorted into units of meaning based on characterizing “what is said” from the individual interviews. Units of meaning with topics within the scope of the study, i.e., describing experiences of sustaining a hip fracture and the path of recovery, were identified and units of meaning with similar topics were grouped into units of significance. Through interpretation of the units of significance, central themes were determined, thereby categorizing and expressing the essence of what was said in the interviews into common themes. Participants’ quotes are presented in the structural analysis, to provide a basis for the developed themes. Apparent emotions are presented in association with the quotes, as non-verbal communication also bears meaning, and further to describe the context in which the statements were made, also reflecting the depth of the data collection.
3. Comprehensive understanding, which comprised a critical interpretation and discussion to reach a further understanding of the text. Through critical reflection, and in relation to relevant literature, the emergent themes were discussed to gain new knowledge and understanding of participants’ experiences. Any discrepancies during the 3 levels of analysis were resolved through consensus between the two authors performing the qualitative analyses.

## Results

In all, 19 individuals agreed to participate. 13 women and 6 men were interviewed at 0.7 to 3.5 years post-fracture. Characteristics for the participants are presented in Table II.

**Table II.** Participant characteristics.

Characteristics	Number of participants = 19
**Age at hip fracture**	
Min-max	32–59 years
Median (IQR)	56 (51–58)
**Marital status**	
Single	5 (26%)
Cohabiting	2 (11%)
Married	12 (63%)
**Level of education**	
Elementary	2 (11%)
Secondary education	11 (58%)
College/University	6 (32%)
**Employment**	
Yes	15 (79%)
No	4 (21%)
**Comorbidity**	
Yes	9 (47%)
No	10 (53%)
**Prior fracture**	
Yes, not hip-related	9 (47%)
Yes, contralateral hip	1 (5%)
No	9 (47%)
**Cause for hip fracture**	
Simple fall/same level fall	8 (42%)
Sports accident	7 (37%)
Fall from height	2 (11%)
Traffic accident	1 (5%)
Work accident	1 (5%)

## Naïve reading and understanding

The apprehension that healthcare and rehabilitation for younger and elderly patients with hip fractures are conducted according to the same standard care plan made the younger participants feel anxious and old from one day to another. Moreover, they felt incapable of actively taking part in their own care and rehabilitation plan. Being forced to act as one’s own health advocate, navigating within a routine-driven and non-receptive healthcare organization, was also described.

Participants described a sense of being treated with ignorance by professional caregivers, who were perceived to have limited knowledge on the participants’ specific condition, when they articulated fears and perceptible symptoms. The participants felt abandoned by those responsible for guiding them on their path of recovery. For our participants, who were all of working age with demands on their physical ability, it was important to receive information on which symptoms were concerning or normal after a hip fracture, and on how they could create optimal conditions for rehabilitation based on their remaining capabilities.

Experiencing strong emotions during the recovery process were described by the participants; shifting from keeping an overly positive façade in front of others to feelings of sadness, helplessness, and disbelief in solitude, struggling to believe in full recovery from the hip fracture. Fear of falling made participants cautious, hesitant, and limited in their surroundings, as well as in social gatherings and new settings, even up to 3.5 years following the hip fracture. Where a participant would once have pushed their limits, restraint was now demanded to listen to the body’s signals and degree of stamina, but also to anticipate any risks that could cause a new fall and potential damage to the injured hip or aggravation of symptoms.

To overcome a hip fracture at a young age required intrinsic motivation to accept any forthcoming physical setbacks, but also to view improvements as a step in the right direction towards regaining their previous abilities and pre-fracture independence. Participants created strategies to motivate themselves to continue the rehabilitation and other activities—to challenge themselves and to prove, not only to themselves but also to friends and family, that they were motivated and had momentum. Attentive and responsive support from healthcare staff was perceived as a vital and decisive factor with potentially significant impact on their path of recovery and residual symptoms, but most participants felt they lacked this advantage.

## Structural analysis

The structural analysis of the interviews revealed that the recovery experience was a painful and protracted process of regaining function, independence, and self-confidence. The fracture brought the participant’s everyday life to a stand-still, creating feelings of weakness, disability, and inability. The interviews revealed different approaches to defying these difficulties and feelings of despair, remaining hopeful and generating motivation for recovery strategies to obtain the pre-fracture level of function.

### Growing old overnight

The participants described a sense of growing old overnight due to the type of fracture they had sustained, especially as friends and family members called their injury an “old people’s fracture”. Similarly, the provided care was executed according to a standard protocol developed from the experience of hip fractures in the older population. Much of the information regarding the fracture and prognosis was given while participants were under the influence of analgesics, leading to problems remembering later during the recovery process. The participants said that upon expressing symptoms, they were ignored and disregarded, receiving contradictory information about the causes of the symptoms and possible methods of relief. The participants’ narrations also depicted the care and rehabilitation as mechanical and numb to the specific rehabilitation needed. They were also told that thanks to their young age, they would heal faster and could expect fewer difficulties during their rehabilitation. The rehabilitation was planned and executed without the involvement of the participant and was perceived as carried through in accordance with a previously defined structure. Participants were prescribed sedative analgesics when discharged, which made them indistinct and non-coherent when returning home and created difficulties in returning to normal routines and relationships with family and friends.

During their hospital stay, participants witnessed the medical staff’s efforts to explain how the fracture would affect their everyday life. The information received from physicians and nurses was perceived as adapted to elderly patients and sometimes as contradictory. *“We are all different, you cannot give me the same instructions as an eighty-year-old” [D1]*.

Participants were guided by a physiotherapist in how to move and what to avoid; however, these appointments were brief and left unanswered questions. Deficient communication left participants in doubt regarding what was valid information. Upon discharge, the participants described an obvious lack of awareness of, and interest in, their home situation and everyday life—for example, how they lived, their ability to receive support with daily chores, how they would manage obligations towards work, family members or close friends, as well as socializing. Participants’ need for transportation was a crucial issue to enable and maintain effective daily routines, but this need was not discussed. Participants were also in consensus regarding the sensation of being abandoned to pursue further rehabilitation on their own, either through municipal care or private caregivers, creating a sense of being forced to act as their own advocate to receive any further rehabilitation without support or assistance with referral from the hospital.

### A person lacking capability

The customization of the participant’s home by the municipal caregivers to permit activities of daily life (removal of thresholds and carpets, elevating the toilet, etc.) further increased the feeling of insignificance and inability to manage on one’s own. Participants found themselves without the capabilities typical of their age group: *“Feeling tired all the time because I do not get the sleep I need because of the pain” [D2].*

Inner age (self-perceived) and outer age (chronological or perceived by others) did not reflect one another. Participants living alone were forced to ask friends or relatives to make daily purchases, which was attended by feelings of self-doubt, shame and inability to cope. The experience of increased load, stiffness and pain from the hip, groin, and surgery incision led participants to feel both discomfort in their limited life, and thankfulness for the support they received. This duality was described as a conflict between needed support and diminished and limited integrity, autonomy, and capability, where participants resisted accepting their need for help.

### Inconsistent emotions and subsequent consequences

Participants described experiencing strong emotions and struggling to confidently believe in a future where they achieved a full recovery from the hip fracture. The path was filled with challenges they had to overcome. Some defined this part of the process as being two individuals: one overly positive and one feeling depressed and hopeless. The participants likewise presented two different personae: one facade that they displayed in front of friends and significant others expressing confidence, and another when they were alone with their thoughts about an insecure and unpredictable future. This latter persona was preoccupied by fear of falling and suffering another fracture, feelings of sadness and entrapment, self-imposed isolation but also external exclusion, as well as frustration and anger towards those feelings of helplessness, weakness, dependence, and frailty.

Pain was explicitly described by most participants, in some cases experienced daily and in others more seldom and less intense. The pain was described as a constant reminder of the fracture, leading to more cautious movements, exhaustion, and dark thoughts of a future with pain and stiffness as fellow passengers. Regardless of incidence or intensity of hip pain, participants described varying levels of fear of falling and doubt in their own body. This led them to create more margins in their life, planning ahead and thinking about what might or might not occur in order to avoid aggravating lingering symptoms. Fear of falling also had negative effects in social contexts, through avoidance of crowds and new, unfamiliar environments, but also of familiar contexts where certain roles and expectations might involve exposure to possible risks. Participants also struggled with the unanswered question of why they broke their hip. *“A low energy trauma hip fracture is an old peoples’ disease—so why me?” [S1].*

To maintain as much normalcy as possible in everyday life during the process of recovery, participants described being forced to overcome adversities and handle reactions from others. The symptoms of the fracture were disguised so as not to be apparent to anyone other than significant others. People around them had difficulty believing in the severity of the symptoms and therefore questioned the participants’ credibility and the seriousness of their limitations. This in turn created shame over the insinuation of over-reacting, leading participants to force themselves to act as others expected them to. Recurrent feelings of growing old, frail, fragile and incapable, which in turn damaged integrity, pride, self-image, and self-worth, were presented in the participants’ narrations. Feeling broken and unmotivated and worrying that the function in their injured leg would never fully return was also expressed.

### Total standstill in midlife

To sustain a hip fracture meant a total standstill in the middle of life. *“My neighbour could walk nicely one month after the operation. I am now one YEAR after the operation and I still have problems even though I am younger. This is embarrassing!” [D5]*. Many participants recounted physical limitations such as fatigue, weak muscles, inability to sit down, stiffness, back pain, and radiating pain from the groin and hip. Ordinary chores were difficult and time consuming due to fear of falling, loss of physical strength, limited leg function, and participants’ mistrust of their own bodies. As a result, some chores were put off to the future. Reduced work capability affected some of the participants, which meant prolonged sick leave or reassignment to other work duties. This in turn created decreased income, a noticeable change and worry for the participant.

Other psychological effects were reduced well-being and feeling depressed, a strong lack of confidence, and uncertainty. Variation in the intensity of the physical symptoms from one day to the next was one of the main factors affecting the participants’ frame of mind.

### Defy despair

Participants had painted a dark and murky picture of the path to recovery with several hindrances, both physical and psychological. However, some experiences also fuelled their motivation and reinforced the will to regain their former condition and bodily constitution. Several aspects in the narrations could qualify as methods to fight the sense of despair and thereby avoid letting stiffness, pain and fear govern their lives.

Participants described actions to strengthen their autonomy and gradually increase the intensity of the rehabilitation without overly burdening the affected hip. These small steps helped them strengthen both internal and external assets, which in turn strengthened their ambition to fully recover. Hope was a crucial ingredient in the recovery process. Setting short- and long-term goals for their rehabilitation amplified this sensation. *“I want to be exactly the same as before the operation but then I understand, I do not have that strength in the leg because it has taken quite a lot of damage. But I want to return to who I was before. I have so many beautiful shoes to use, ones with really high heels. They have been my motivation to get better (laughs), because I decided I will use them again (laughs)” [S2].*

Some participants recounted several strategies to generate the strength to complete the exercise sessions. Decisive factors in completing the rehabilitation were, according to the participants, early mobilization and the use of aids in their home to preserve strength to be able to attend rehabilitation sessions. Additional strategies to maintain progress included stopping to rest when feeling overexerted, keeping a positive attitude and maintaining physical activities at home between exercise sessions. Changing routines could also significantly help reduce stress and increase the sense of autonomy.

### Returning to normal

Recovery after the hip fracture was described as a protracted process. *“I think it has taken a long time to get back to normal. And, well, I am not quite sure that I actually am fully back to normal … But now is maybe the new normal” [S3].*

Continuous rehabilitation required motivation to persist. Belief in improvement, strength, and endurance to actively partake in scheduled activities or meetings with physiotherapists were expressed as crucial for the participants. Some recognized procrastination and used excuses to avert the exertion, avoiding the overwhelming reality of the lengthy path to full recovery. Some participants explained that they had the will, but their body refused. Others told themselves that rehabilitation must work, which increased their motivation to continue fighting and not give up.

Participants emphasized the need to find methods to increase the motivation for recovery, even when the path felt dark. Some maintained social networks and pointed to this as an important part of their rehabilitation pathway. More objective determining factors to preserve motivation were housing, civil status, understanding employers and continuous feedback from physiotherapists with a program based on the person’s abilities and strength. The participants experienced that being in good physical shape before the accident determined their level of motivation and odds of a successful recovery by contributing better capacity and ingenuity of finding ways forward. Additionally, the perceived level of competence and professionalism of the physiotherapist made a major difference for the participants, as did increased trust in their own body and their immediate surroundings’ understanding of the long rehabilitation process.

## Comprehensive understanding and discussion

The main finding is that the participants experienced significant challenges in their daily lives, even up to 3.5 years after the fracture. They also expressed a desire for individually targeted rehabilitation and support of their needs, and some described feeling neglected by the healthcare system.

### Healthcare staff-imposed challenges in recovery immediately after injury

Encounters with healthcare staff matter to patients. Our participants disclosed a sense of growing old overnight, due in part to the type of fracture but first and foremost due to the way the staff treated them. The feeling of standardized and mechanical care and rehabilitation without patient involvement, and the fact that participants felt ignored, disregarded and that they received contradictory information, support previous suggestions that awareness of younger patients’ specific needs for recovery must be acknowledged (Janes et al., 2018).

### Lingering challenges

Pain was explicitly described by most participants. Lingering pain years after the hip fracture in younger patients was previously described by Swiontkowski at al (Swiontkowski et al., 1984) almost four decades ago. This suggests that outcomes have not improved sufficiently with time, despite other improvements in healthcare services.

Fear of falling was a prominent reality for our participants, as previously reported among younger patients (Janes et al., 2018). In older adults, associations with poorer functional recovery and lower quality of life have been found (Bower et al., 2016; van der Vet et al., 2021; Visschedijk et al., 2010). Fear of falling is an important factor to address during the care and rehabilitation after hip fractures, and awareness is a prerequisite for prevention of any negative effects.

A general wish among the participants was to return to their normal, pre-fracture state. Several studies of older patients described sustaining a hip fracture as a “lifebreaking event” because of the multidimensional consequences the injury has on their everyday life, both psychological and social (Jensen et al., 2017; Zidén, Frandin, et al., 2008; Zidén, Wenestam, et al., 2008).

Recurrent feelings of becoming old, frail, fragile and incapable were presented, which in turn damaged integrity, pride, self-image, and self-worth. A previous qualitative study on patients under age 60 with hip fractures reported psycho-social impact to be present up to 10 years following the fracture (Janes, 2016), supporting our finding of lingering implications and highlighting the need for long-term follow-up of results.

The hip fracture was described as bringing life to a total standstill. Some of the participants could not satisfactorily perform their work obligations, which meant prolonged sick leave or modified tasks. This supports previous suggestions of potential economic implications due to a hip fracture in individuals of working age (Holt et al., 2008).

### Factors influencing recovery

Standardized plans for care and rehabilitation after hip fractures are based on scientific evidence but were regarded by our participants as rigid and not individually customized. The ideal care might also involve a more holistic view of the patients as individuals, with their specific needs being met. Participants in our study reported that individually targeted rehabilitation and support of needs contributed greatly towards their recovery, and those who did not receive it expressed a lack of it. Similar needs were identified by a study on hip fractures in all ages that found less than one third of the patients considered their rehabilitation to be adequate (Hansson et al., 2015). This indicates that there is obvious room for improvement regarding support of recovery.

Other factors we found to encourage recovery were hope and belief in improvement, support from family and friends and understanding employers. It appears that social support is equally important to our younger participants as it is to older adults after a hip fracture (Beer et al., 2021). Difficulty appreciating the severity of lingering symptoms and limitations by the outside world has also been reported in the UK (Janes, 2016). Our study participants emphasized the need to find ways to increase and maintain motivation.

### Evaluation of outcome

Traditionally, reports of the outcome of orthopaedic interventions as successful or failure have been determined by surgeons, focusing on complications or re-operation rates (Ashby et al., 2009). These outcomes are important and quantifiable, but absence of complications or re-operation does not necessarily equal a successful recovery from a patient’s perspective (Hsiao & Fraenkel, 2017; Waljee et al., 2016).

Recommendations on reporting hip fracture outcomes important to patients include radiographic, clinical and functional outcomes (Sprague et al., 2015). Additionally, a more patient-centred core outcome data set, including presence of hip-related pain and limping; level of return to daily life activities, work, sports and leisure activities; and assessment of health-related quality-of-life and objective functional performance have been suggested (Rogmark et al., 2018).

Through the present study, we add the explicitly patient-centred outcome of the individual’s experience of sustaining a hip fracture by illuminating physical, psychological, and social perspectives. Adding a psychosocial assessment might be of value in future evaluation of outcomes following hip fracture.

### Future support in recovery after hip fractures

The findings of lingering physical and psychosocial implications suggest a need for continuous long-term support of patients sustaining a hip fracture. The physical and psychosocial factors enabling recovery are similar in both older and younger patients (Janes et al., 2018). Our participants expressed the same thoughts on recovery as those reported in a qualitative systematic review of hip fracture recovery in older patients (Abrahamsen & Nørgaard, 2021). This indicates that chronological age might be a poor measure to predict recovery or guide healthcare support of recovery. On the other hand, it has been proposed that the higher demands of a younger and more active individual, e.g., at work or in physical leisure activities, can be harder to fulfil (Rogmark et al., 2018; Sprague et al., 2015). Full return to a pre-injury state of mobility and function seems difficult to reach for all patients, and psychosocial implications affect patients years after injury (Bertram et al., 2011; Ekegren et al., 2016; Hansson et al., 2015; Janes, 2016; Rogmark et al., 2018).

Perhaps recovery from injury should not be defined as a return to the previous self-perceived definition of oneself, as for some this appears to be an impossible target. In a qualitative study of patients who survived life-threatening accidents, it was reported that a redefinition of oneself was crucial to self-preservation (Morse & O’brien, 1995). This redefinition may also be of value for patients who have suffered hip fractures—taking previous and recent experiences and the abruptly developed new life situation after injury into account—focusing on expectations, aspirations and aims from both physical and psychosocial perspectives, with support from health care services.

Patients must be informed of the lengthy rehabilitation process, and rehabilitation should be tailored to the individual (Proctor et al., 2008; Welch et al., 2020). This study, as well as other studies, have identified that this individualized care is lacking (Eastwood et al., 2002; Röding et al., 2003).

Our results suggest provision of tailored and alternative pathways of rehabilitation, including support of the patient’s redefinition of self after suffering a hip fracture. Healthcare services should be equipped to provide adequate support for the recovery of all patients, not only standard geriatric hip fracture rehabilitation. From the point of view of both the patient and society, future research must identify the subgroups of patients with hip fractures who do and do not recover, to better understand what can be expected after the injury.

### Limitations and strengths

Our participants were purposively sampled from a larger cohort and they represent a broad sampling of characteristics and a variety of experiences. The difference in time from the hip fracture to the interview might introduce a recall bias with a longer time, but it also gave the possibility to explore a variation in the participants’ experiences with time. The impact of a possible recall bias was not possible to estimate, however, we aimed to illuminate patients’ experiences after sustaining a hip fracture, not to provide a complete documentation of all patients’ experiences at exact time points. As a sample of experiences, our participants’ contributions are valid, highlighting a variety of aspects of recovery that matter to patients.

We explored the participants’ experiences through interviews, in which they expressed their notions of what was important for their recovery. The qualitative method enabled an improved understanding of aspects of recovery after hip fracture that are significant to patients. Our results add to—and support—a small but emerging body of knowledge, suggesting that our findings are transferable to patients with hip fractures in other high- and middle-income countries.

The collection and analysis of data were performed in accordance with the method of phenomenological hermeneutics, following three welldefined methodological abstraction levels, which strengthens the trustworthiness of the study in reproducibility (Lindseth & Norberg, 2004).

## Conclusion

The lived experience of sustaining a hip fracture in adults under 60 years includes challenges in everyday life, even years after the injury. Lingering pain and feelings of weakness, disability and physical inability were expressed by participants. The provided care and rehabilitation were perceived as adapted to elderly patients, not to the needs of younger individuals. In perspective, other pathways of care and rehabilitation, including improved information, are suggested in order to meet the diverse demands of all patients with hip fractures.

## Supplementary Material

Supplemental Material
