# Supplementary material for: Lingering challenges in everyday life for adults under age 60 with hip fractures – a qualitative study of the lived experience during the first three years
Source: Int J Qual Stud Health Well-being. 2023 Mar 17;18(1):2191426. doi: 10.1080/17482631.2023.2191426 (PMC10026814; doi:10.1080/17482631.2023.2191426)
Supplement: Supplemental Material [file ZQHW_A_2191426_SM1031.docx]

**Supplement table 1** Example of data interpretation in the structural analysis

| **Unit of meaning** | **Unit of significance** | **Theme** |
| --- | --- | --- |
| *“We are all different, you cannot give me the same instructions as an eighty-year-old.”* | *Being different and not like an eighty-year-old.* | *Becoming old overnight*. |
